# First Complete Coding Sequence of a Spanish Isolate of Swine Vesicular Disease Virus

**DOI:** 10.1128/genomeA.01742-15

**Published:** 2016-03-03

**Authors:** Ángela Vázquez-Calvo, Juan-Carlos Saiz, Francisco Sobrino, Miguel A. Martín-Acebes

**Affiliations:** a Centro de Biología Molecular Severo Ochoa (CSIC-UAM), Cantoblanco, Madrid, Spain; b Departamento de Biotecnología, Instituto Nacional de Investigación y Tecnología Agraria y Alimentaria (INIA), Madrid, Spain

## Abstract

Swine vesicular disease virus (SVDV) is a porcine pathogen and a member of the *Enterovirus* genus within the *Picornaviridae* family. The SVDV genome is composed of a single-stranded RNA molecule of positive polarity. Here, we report the first complete sequence of the coding region of a Spanish SVDV isolate (SPA/1/'93).

## GENOME ANNOUNCEMENT

Swine vesicular disease virus (SVDV) is a member of the *Enterovirus* genus within the *Picornaviridae* family. SVDV is the etiological agent of a highly contagious disease of pigs (SVD) and is closely related to the human pathogen coxsackievirus B5 (CVB5) (1). The SVDV genome is composed of a single-stranded RNA molecule of positive polarity. This RNA molecule of about 7,400 nucleotides in length encodes a polyprotein in a single open reading frame that is flanked by two noncoding regions (NCRs) located at the 5′ and 3′ ends of the genome. This polyprotein is translated using an internal ribosome entry site (IRES) and is processed into the mature viral proteins (2, 3). In this report, we sequenced the complete coding region and 164 nucleotides of the 5′ NCR of the SVDV SPA/1/'93 strain using reverse transcription (RT)-PCR. The virus was isolated from a pig infected in the outbreak that occurred in 1993 in Lerida, Spain (4) and propagated in IBRS-2 cells (seven passages) and the viral RNA was extracted from supernatants of infected cell cultures by using TRI Reagent (Sigma). cDNA was synthesized by reverse transcription of viral RNA using murine leukemia virus reverse transcriptase (Roche) and the oligonucleotide primers indicated below. cDNA was amplified by PCR using specific oligonucleotide primers and BioTaq DNA polymerase (Bioline) supplemented with Expand high-fidelity polymerase (Roche) for proofreading activity (8/1). Oligonucleotide primers used for RT-PCR targeted conserved sequences on the SVDV genome, as revealed by the alignment of the complete genome sequence of different SVDV isolates and two isolates of CVB5 (GenBank accession numbers of the sequence selected: EU151454.1, AF268065.1, EU151461.1, D16364.1, D00435.1, AY429470.1, EU151450.1, AY875692.1, and AF114383.1). The suitability of these oligonucleotide primers for DNA amplification was verified using the PCR Primer Stats from Sequence Manipulation Suite. The selected oligonucleotide primer pairs were 1S (GGAACCGACTACTTTGGGTGT), 1AS (GTTGTGCCTGAACATGTTGTCC), 2S (CGTTGGTGTTGGTAATCTAAC), 2AS (TGAGTGGTAATTCTTCACATGCC), 3S (CCAGTCAACTCGGAGTCCATC), 3AS (CTTCCCACACACAGTTTTGCC), 4S (GGGTAGAACAGATATAACAACC), 4AS (CCGTACCTCACCTATCGGTAG), 5S (GGGATCCCTATGGCTGAAAG), 5nS (GTTGCCACGCCATGCTAAAC), 5AS (GTTGGGAAATTGTACATGAGCATTC), 6S (GGGTGACTGACTACGGGTTC), and 6AS (GTTCGGTTCATGCGGTAGGG). PCR products were purified with Wizard SV gel and a PCR cleanup system (Promega), quantified by UV spectrophotometry using Nanodrop equipment (Nanodrop Technologies) and sequenced by automatic DNA sequencing (Macrogen). DNA sequences were confirmed by at least two independent sequencing reactions performed using the different oligonucleotide primers. Sequences were assembled and analyzed using Seqman II 5.01 from the DNASTAR Lasergene package.

The coding sequence of the Spanish SVDV isolate SPA/1/'93 was compared with other complete coding sequences from different isolates that varied geographically and temporally using Clustal 2.1 (GenBank accession numbers: EU151454.1, EU151455.1, D16364.1, D00435.1, KF963275.1, KT284996). This analysis showed that SVDV SPA/1/'93 shared a higher degree of sequence identity (over 98%) with contemporaneous Italian isolates (EU151454.1, EU151455.1). The sequence data of the SVDV isolate determined in this study will help future research on the epidemiology and molecular biology of this porcine pathogen.

### Nucleotide sequence accession number.

The genome sequence of the SVDV SPA/1/'93 has been deposited in GenBank under the accession number KU291213.
